# A Task Force Against Local Inflammation and Cancer: Lymphocyte Trafficking to and Within the Skin

**DOI:** 10.3389/fimmu.2018.02454

**Published:** 2018-10-24

**Authors:** Fanny Lafouresse, Joanna R. Groom

**Affiliations:** ^1^Divisions of Immunology and Molecular Immunology, Walter and Eliza Hall Institute of Medical Research, Parkville, VIC, Australia; ^2^Department of Medical Biology, University of Melbourne, Parkville, VIC, Australia

**Keywords:** lymphocyte trafficking, skin, adhesion molecule, chemokine, melanoma

## Abstract

The skin represents a specialized site for immune surveillance consisting of resident, inflammatory and memory populations of lymphocytes. The entry and retention of T cells, B cells, and ILCs is tightly regulated to facilitate detection of pathogens, inflammation and tumors cells. Loss of individual or multiple populations in the skin may break tolerance or increase susceptibility to tumor growth and spread. Studies have significantly advanced our understanding of the role of skin T cells and ILCs at steady state and in inflammatory settings such as viral challenge, atopy, and autoimmune inflammation. The knowledge raised by these studies can benefit to our understanding of immune cell trafficking in primary melanoma, shedding light on the mechanisms of tumor immune surveillance and to improve immunotherapy. This review will focus on the T cells, B cells, and ILCs of the skin at steady state, in inflammatory context and in melanoma. In particular, we will detail the core chemokine and adhesion molecules that regulate cell trafficking to and within the skin, which may provide therapeutic avenues to promote tumor homing for a team of lymphocytes.

## Introduction

The skin, the body's largest organ with its 1.8 m^2^, represents our first line of defense and comprises a wide diversity of innate and adaptive immune cells. It is a complex organ as revealed by its structural organization in layers and by its multiple physiological functions. Indeed, while the skin provides a barrier protection from external environment, it further acts as a physical sensor and a temperature and hydration regulator by communicating with the outside environment. At homeostasis, lymphocytes constantly traffic to and within this complex environment, patrolling for pathogen invasion, inflammatory conditions and malignancy development. Loss of skin immune cell integrity can lead to diverse local diseases like atopic dermatitis, psoriasis, or tumor development (1, 2).

This review intends to map lymphocyte trafficking routes to and within the skin at homeostasis. Furthermore, we point out how lymphocyte trafficking responds to threats and we finally discuss how a better understanding of lymphocyte recruitment, specific location within the skin and interacting partners is essential to identify targets that may be manipulated for therapeutic benefits in cancer.

## Skin: home of a plethora of cells within a complex structure

**Figure 1:**
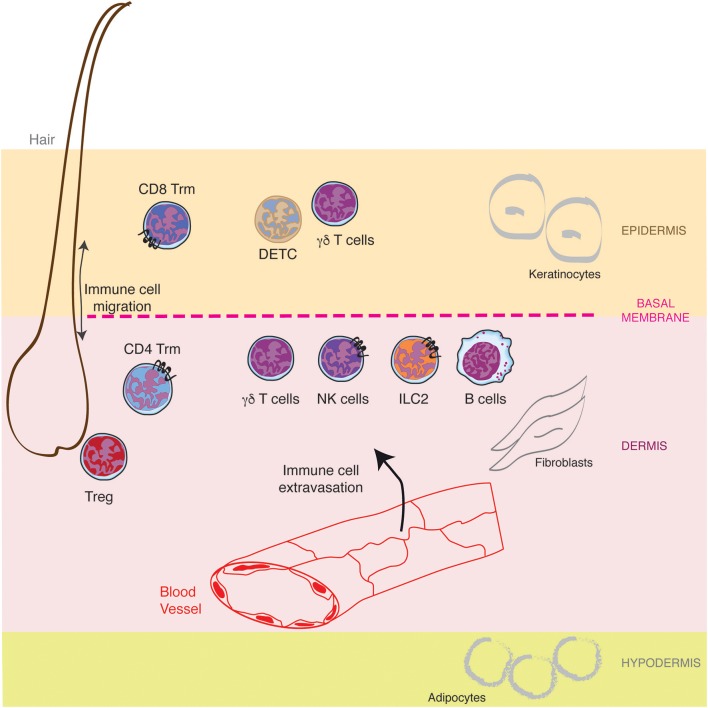
Lymphocytes specific location in healthy skin. The skin is composed of a succession of three different layers, the epidermis, the dermis and the hypodermis populated by resident lymphocytes and lymphocytes recruited from the blood circulation through the cascade of molecular interactions. Furthermore, hair follicles represent a specific niche for some lymphocytes.

Mammalian skin is composed of a succession of three different layers, the epidermis, the dermis and the hypodermis, comprising nerves, blood vessels, glands, and hair follicles (Figure 1). The outer epidermis is a stratified squamous epithelium (the stratums: corneum, granulosum, spinosum, and basale), mainly populated by keratinocytes (80%), melanocytes, Langerhans cells, and Merkel cells (nerve-ending cells). Below the epidermis, separated by a basement membrane, the dermis is a thicker layer which home various cell types (fibroblasts, immune cells) into a collagen-rich complex matrix. The collagen fibers (thin in the upper stratum papillare and thick in the lower stratum reticulare) offer a structural framework to host cells. Underneath, the hypodermis is an adipocyte rich region. While the epidermis is not vascularized, the dermis is innerved by lymphatic and blood vessels both of them playing important role in outcoming and incoming trafficking of cells, small molecules and pathogens (Figure 1). The dermal lymphatic system represents a major conduit for pathogens and immune cells from the skin to the draining lymph node. This has been recently reviewed elsewhere (3, 4) and will not be described here further.

The skin harbors a wide variety of immune cells including macrophages, mast cells, dendritic cells, and lymphocytes (3). Some of these subsets seed the skin during embryonic development and become skin resident cells while others continuously recirculate or are replenished from circulating bone-marrow precursors. Our review focuses on innate and adaptive lymphocytes, including T lymphocytes, B lymphocytes, and innate lymphoid cells (ILC). These cells either reside in the skin or are recruited to the skin from the blood circulation at homeostasis and/or following inflammatory signals.

While, it was first assumed that healthy human skin was devoid of B lymphocytes (4), a heterogeneous population of B cells (large B-cells and IgM^high^ B-1-like B cells) has been observed in sheep dermis where their expression of MHC-II and CD80/86 was slightly increased compared to lymph node B-cells (5). Recently, immunohistochemical staining revealed the presence of CD22^+^ B cells in around 30% of healthy human skin samples (6). These cells were mature IgG antibody-expressing B cells distinct from the circulating B cells suggesting that class switched and somatic hypermutated B cells localize to the skin at homeostasis. As opposed to B lymphocytes, T lymphocytes are highly represented in the skin. In healthy individuals, it has been estimated that 20 billion of T lymphocytes populate the entire skin surface, strikingly, this is almost twice the number of T lymphocytes found in the total blood circulation (7). Most of the cutaneous αβ T lymphocytes are resident memory T cells. Both memory CD4^+^ and CD8^+^ T cells were observed in the skin with 20–60% of murine cutaneous CD4^+^ T cells being Treg (8, 9). Unconventional γδT lymphocytes, dendritic epidermal γδ T cells (DETC, Vγ5 Vδ1) and dermal γδ T cells (γδT17 cells) are also skin resident cells under homeostatic conditions. These populations participate in early stage of immune response (9, 10). In human skin, γδ T cells represent <10% of the dermis and epidermis total T cells whereas in mice epidermis, DETC alone constitute 90% of the total T cells (3, 11). γδ T cells recognized self and conserved motifs of microbial-derived antigens.

ILC are non-T, non-B lymphocytes, characterized by a lack of myeloid and dendritic cell phenotype markers and by a lymphoid morphology. Recently, a new nomenclature has been proposed classifying ILC in 5 subgroups: ILC1, ILC2, ILC3, conventional natural killer cells (NK) and lymphoid tissue inducer cells (LTi) (12). While ILC1-3 and NK populations have been observed in healthy human and mouse skin, ILC2 represents more than 90% of them in mice skin (13). In terms of function, ILC2 are an important source of type-2 cytokines and has been associated with allergic inflammation in the skin. Further, healthy human skin is populated by both immature CD56^bright^ CD16^−^ and mature CD56^dim^CD16^+^ NK cells (10, 14). Their frequency varies from 1 to 10% of total CD45^+^ leucocytes between studies, those cells being mainly enriched in the blood circulation. NK cells are cytotoxic to virus-infected cells and tumor cells and cytokine producers.

In addition to immune cells, the skin is also colonized by resident microorganisms (bacteria, fungi, parasites, viruses), feeding on corneocyte debris and sebum. The skin microbiome is essential not only to prevent the development of pathogenic microbes for the organism, but also to educate the immune system (15) and finely tune immune cell functions (16). Recently the skin transcriptome of germ-free mice has been compared to the one of specific pathogen mice (microbiota positive), highlighting more than 2,800 genes modulated in the skin by the microbiota (17). Particularly, 37 genes, including chemokine family gene *Ccr2, Ccr5, Ccl5, Ccl6, Cxcl8, and Cxcl9*, were upregulated during microbial colonization in the skin and the gut (17, 18). While the Meisel et al. study focussed their bioinformatic analysis to genes co-regulated in the gut and skin microbiota, further datamining of this work would highlight genes specifically regulated in the skin. Indeed, these tissue specific genes may represent interesting therapeutic targets to balance immune cell recruitment in a site-specific manner.

## Lymphocyte entry within the skin at steady state: a selective system

### The cascade of molecular interactions for circulating lymphocyte recruitment

**Figure 2:**
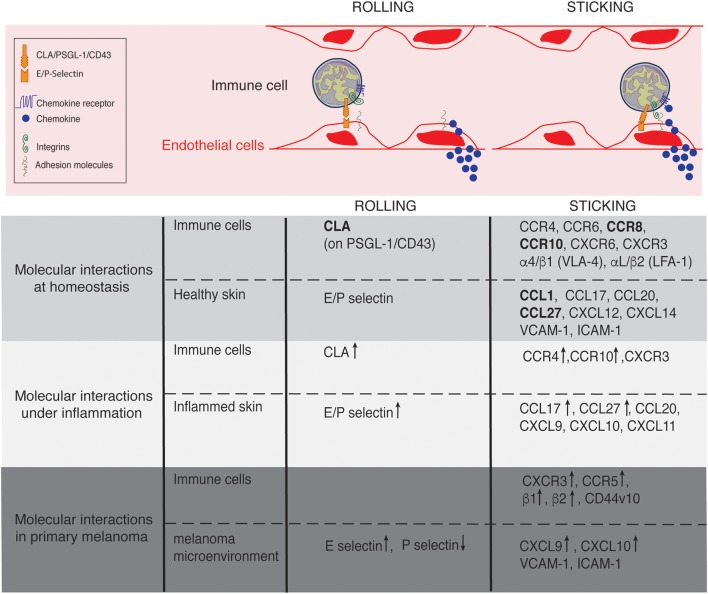
Lymphocyte trafficking into the skin at homeostasis, under inflammation and in primary melanoma. Molecules involved in the rolling and sticking (firm adhesion) steps of lymphocytes recruitment at homeostasis, under inflammatory conditions and in primary melanoma are listed on the figure. Of interest, most of the molecules are shared between conditions. ↑ and ↓ indicate up- and down- regulated expression, respectively in comparison to homeostasis. Skin-specific molecules are in bolt.

Postcapillary venules composed by a flat endothelium represent the main gateway for lymphocyte recruitment in non-lymphoid tissues. In contrast to other non-lymphoid tissues where leukocyte recruitment is rare or inexistent in the absence of inflammatory stimuli, a constitutive afflux of lymphocytes occurs at steady state in the skin (19, 20). This afflux remains low when compared to the maestro in terms of recruitment: the lymph node high endothelial venules (21, 22). However, similar to that described in lymph nodes, lymphocyte entry in the skin is finely regulated by a cascade of interactions mediating immune cell rolling, adhesion and extravasation (23) (Figure 2). The initial capture of circulating lymphocytes by endothelial cells is achieved by the interaction between members of the selectin family with their ligands, slowing down flowing lymphocytes. Real time intravital microscopy imaging first shed the light on cell rolling, recognizable by a catch bound cell displacement along vessel walls (19, 24). Selectins have a rapid association and dissociation rate constant (K_on_/K_off_) and a high tensile strength, supporting the observed transient interactions (25). The selectin family which comprises 3 members (E-selectin, L-selectin, and P-selectin), bind to sialyl-Lewis^x^ (sLeX) carbohydrates (26), fucosylated by the fucosyltransferase (FucT)-IV and FucT-VII (20, 27). The rolling phase allows lymphocyte to examine the vessel wall for chemoattractant signals (chemokines and lipids). In turn, chemokine receptor stimulation together with the hemodynamic shear force of blood flow, induce integrin conformational changes from a low-affinity conformation to a high-affinity conformation, within milliseconds, allowing cell firm adhesion on the endothelium (28, 29). Integrins are linked to the actin cytoskeleton by binding to cytoskeleton-associated protein such as talin, inducing lymphocyte morphological changes (30). Lymphocytes take up a polarized morphology forming a leading edge and an uropod to transmigrate between endothelial cells (paracellular transmigration) or through them (transcellular transmigration) (31). The cascade is broadly mechanistically identical for all immune cell recruited across all sites and under physiological or pathological context (23). However, the specificity of the process is controlled by the diversity of the selectin, integrin and chemokine molecules expressed by a tissue and the expression of the matching receptors or ligands on circulating cells (28). Furthermore, as each step of the cascade condition the next one, the combination adds extra specificity to immune cell recruitment at a particular site of the organism.

### Skin-associated selectins, chemokines, and integrins

The anatomical site of immune cell recruitment is not random but strictly determined by the expression of a tissue-specific set of adhesion molecules and chemokine receptors (Figure 2). Compared to other tissues, the set of adhesion molecules/chemokines expressed in the skin (Table 1) is particularly unique.

**Table 1:** Expression of adhesion molecules, chemokines and chemokine receptors at the surface of the indicated cells at steady state, under inflammation and in melanoma.

	**Steady state**				**Inflammation**						**Cancer**			
	**Selectin**	**Integrin ligands**	**Chemokines**	**References**	**Selectin**		**Integrin ligand**	**Chemokines**		**References**	**Selectin**	**Integrin ligands**	**Chemokines**	**References**
Skin endothelial cells	E-selectin (low), P-selectin		CXCL12, CXCL14, CCL1, CCL20, CCL17	(20, 32–35)	P-selectin, E-selectin			CCL17↑, CCL27↑ (displayed, not produced)		(36–38)	P-Selectin↓	VCAM-1↓, ICAM-1/2↓, CD34↓	CXCL9↑, CXCL10↑	(39), (40), (41), (42), (43), (44), (45), (46)
Keratinocytes			CCL27	(47)				CCL27↑, CXCL9, CXCL10, CXCL11, CCL20		(38)				
Other dermal cells			CCL1 (Langerhans cells, Melanocytes)	(34)										
	**Steady state**				**Inflammation**						**Cancer**			
	**Selectin Ligands**	**Integrins**	**Chemokine receptor**	**References**	**Selectin**	**Selectin ligands**	**Integrins**	**Chemokine receptor**	**References**	**Selectin**	**Selectin ligands**	**Integrins**	**Chemokine receptor**	**References**
T lymphocytes	CLA		CCR8, CCR4	(7)	L-selectin	CLA↑		CCR10↑, CCR4↑	(36, 48, 49)			CD44v10	CXCR3, CCR5	(50), (51), (52), (53), (54)
Trm		CD103, CD69, CD49d	CCR8, CCR10, CXCR3, CXCR6	(55–59)				CXCR3 (Trm precursors)	65					
Treg	CLA	CD103, CD69	CCR4, CCR6	(8, 60–62)		CLA		CCR4	(63)					
ILC2	CLA	CD103	CCR4, CCR10	(64, 65)										
NK	CLA		CCR8	(10, 66)				CCR5, CCR6, CXCR3, CCR8, CXCR1	(67, 68)				CXCR3	(69, 70)

*↑ and ↓ indicate up- and down-regulated expression in comparison to steady state condition*.

Three C-type lectins composed the highly conserved Selectin family: the L-Selectin (expressed on leukocytes and hematopoietic stem cells) and the vascular selectins, E-Selectin (expressed on endothelial cells) and P-Selectin (expressed on endothelial cells and platelets) (29). Selectins are type I membrane glycoproteins with an N-terminal C-type lectin domain, an EGF-like motif, a transmembrane domain and a short cytoplasmic tail. Skin endothelial cells constitutively express low level of E-selectin allowing leukocyte rolling at low velocity whereas P-selectin determines the rolling frequency (20). L-selectin is not expressed by endothelial cells but it is expressed at the surface of some subsets of leukocytes. However, it has been shown that L-selectin is not involved in immune cell rolling at homeostasis.

The chemokine family comprises about 50 small chemokine ligands recognized by ~20 seven-transmembrane, G-protein-coupled receptors (71). Most of the chemokine receptors have several ligands and *vice-versa*. We have previously discussed the implications of a such apparent redundancy, illustrated with CXCR3 and its three ligands CXCL9, CXCL10, and CXCL11 (72). Originating from the Keystone symposium on Chemokines and Chemotactic Receptors in 1999, our current nomenclature of the chemokine and chemokine receptor is based on the arrangement of the two N-terminal cysteine residues forming a disulfate bond and defines 5 groups: CXC, CC, X(C), CX3C, and CX (73). Chemokines can also be segregated in two functional groups: the homeostatic chemokine are constitutively expressed by specific tissues, they retain immune cells within tissues and are involved in their survival and proliferation while the inflammatory chemokine are rapidly induced upon environmental changes allowing a massive on-site recruitment of immune cells. Interestingly, homeostatic chemokines are relatively ancient in the evolution and really well conserved between species compared to the inflammatory chemokines which have rapidly evolved (74). Consequently, homeostatic chemokines represent appealing therapeutic targets, because of the likely transferable knowledge between mouse and human studies and tissue tropism. Chemokines are small molecules which diffuse from their production site forming a gradient that can be sensed by polarized chemokine-receptor expressing lymphocytes. Chemokines can be immobilized on negatively charged scaffold proteins, like heparan sulfates by binding though their positive amino acid (75). The retention and immobilization of soluble chemokines is thought to establish gradients, allowing immune cells to sense differing chemokine concentrations to establish directionality. Skin endothelial cells constitutively display chemokines that are either produced by themselves or passed to them to be presented. This include CXCL12 (SDF-1) (32), CXCL14 (BRAK) (33), CCL1 (34), CCL20 (35), and CCL27 (CTACK) (47). CCL1 and CCL27 are the most skin-associated chemokines and by consequence represents interesting targets for controlling immune cell trafficking within healthy skin. CCL27 was firstly named CTACK for Cutaneous T cell Attracting ChemoKine because of its constitutive and unique expression by cutaneous keratinocytes. CCL27 can be further induced by TNF-α and IL-1β and binds CCR10 (76). CCL1 is constitutively expressed by dermal vessels, epidermal melanocytes and Langerhans cells and binds CCR8 (34).

Integrins are composed of one α- and one β-subunits of noncovalently-linked type I transmembrane glycoproteins (77, 78). 18 α-subunits and 8 β-subunits forming 24 distinct heterodimers has been described in mammals (79). On resting cells, integrins harbor a bent (inactive) conformation offering a low binding affinity for its ligands, that is switched to an extended (active) conformation of high binding affinity. VLA-4 (α4β1), α4β7, LFA-1 (αLβ2) are the major integrins expressed by lymphocytes however, none skin-specific ligands for integrins has been reported under homeostatic conditions in the skin.

### Expression of selectin-ligands, chemokine receptors, and integrins by cutaneous lymphocytes

In the skin-draining lymph nodes, effectors and memory populations receive “imprinting” signals from dendritic cells (DC) to instruct their specific migration to the skin (80). Lymph node effector CD4^+^ and CD8^+^ T cell upregulate P-selectin ligands expression and downregulate the gut-associated integrin α4β7 addressing them to the skin (81, 82). One mechanism of such “imprinting” in human involves vitamin D3 metabolization to its active form by DC, leading to an upregulation of CCR10 expression by lymph node T cells, which guides them to the skin (83). More recently it has also been shown that skin Langerin^+^ CD11b^+^ migratory DC induce E-selectin expression on naïve CD8^+^ T cells upon topical immunization (84). Circulating lymphocytes subsets expressing a matching set of receptors/ligands for the skin are constitutively recruited (Table 1). In healthy skin, T lymphocytes express high level of the cutaneous lymphocyte antigen (CLA), a ligand for E- and P-selectin, correlating with high E-selectin-binding activity (7). CLA is an inducible sLeX carbohydrate epitope found on PSGL-1 and CD43 (85, 86). Cutaneous T lymphocytes also largely express CCR4 and CCR6, half of them are positive for CCR8 and CXCR6 expression and about 20% are CXCR3^+^ (7). By contrast, in the circulation, only around 40–60% of CLA^+^ T cells express CCR4, CCR6, CCR7, or L-selectin and <2% CCR8, CXCR6, or CXCR3 (7). Combined, this work highlights the predominant role of CCR4 and CCR6 for T cell entry into skin.

Only a low proportion of Treg migrate to the skin under homeostatic conditions in adult (63). However, an important wave of Treg migrate to the skin between day 6 and day 13 after birth in mouse in a CCR6 dependent manner seeding this organ (8). Presumably, this homeostatic recruitment into the skin, coincides with and instructed by the seeding of the cutaneous microbiota after birth, however, this has not formally been tested. Most of human circulating Treg are CLA, L-selectin, CD11a, CCR4, CCR6, and CXCR4 positive (>70%) (60). Treg play a critical role in controlling tissue homeostasis in the skin as CCR4 deficiency in murine Treg lead to impaired Treg homing to the skin and development of severe skin inflammatory disease (61).

Human cutaneous ILC2 expressed CLA, CCR4, and CCR10 (64). Despite expressing specific set of adhesion molecules/chemokine receptors, which theoretically allow their recruitment into the skin from the blood circulation, ILC2 recruitment is still heavily debated and need to be further investigated (as discussed below). Most of the skin NK studies have been conducted in the context of inflammatory disorders or cancer while cutaneous NK cells at homeostasis have been poorly documented in comparison to T cell trafficking. Yet, it has been shown that both human and mouse NK cells mainly locate in skin dermis and that some human cutaneous NK cells expressed CLA and CCR8 but do not expressed CCR7 (10, 66). However, a complete phenotyping of these cells under homeostatic condition is so far lacking.

Recently, it has been observed that a small proportion of circulating B cells express CLA in healthy individuals (6). Sheep skin B cells express α4, β1, and β7 integrin and 16% of them also express E-Selectin ligands (5). In *in vitro* assays, these B cells migrate toward CCL20 but not CCL17, CCL28, and CCL1, however the minimal required combination of receptors required for cutaneous B cell recruitment is yet to be determined.

## Lymphocyte residency in the skin at steady state

A growing number of resident cutaneous lymphocytes has been identified including tissue-resident memory T cells (Trm), Treg but also unconventional T cells and ILCs. Trm provide superior protection against infection and recent studies indicate that they play a crucial role in cancer immunosurveillance (87–89). Indeed, Trm has been associated with a good prognostic value in lung cancer, ovarian carcinoma and more recently in breast cancer (88, 90–92). Furthermore, Trm were required for the efficacy of cancer vaccine in cutaneous melanoma and other cancer (88, 93). These studies underlie the crucial need to better characterize skin-resident populations as their manipulation via therapeutic intervention has clinical potential. The phenotype of the resident populations is highly heterogenous between tissue of residency and the nature/history of pathogen infection, underlying the pivotal influence of the environment on resident-lymphocyte phenotype and functions. However, recent studies had revealed a common transcriptional program for Trm which is distinct from the one of circulating T lymphocytes (94–96). Hobit and Runx3 transcription factors are known master regulators of tissue residency (97). Trm downregulate genes implicated in tissue egress such as *S1pr1, S1pr4, S1pr5* and upregulate the chemokine *Xcl1* and chemokine receptor *Cxcr6* genes. Trm were first discovered in the skin in graft models (98). Indeed, symptomless skin from patients with psoriasis engrafted onto immunodeficient mice gave rise to active skin lesions, thereby demonstrating the presence of a pathogenic population residing in healthy skin. Broadly, Trm lack the lymph node homing molecules CCR7 and CD62L and express CD103 (also known as the integrin α-E subunit), CD69 and CD49d (VLA-1) although these markers do not strictly identify Trm, some of them being either CD103^+^ or CD69^+^ or negative for both markers (55, 56). In mice, the dominant pool of Trm, CD8^+^ CD103^+^ cells locate in the epidermis while in human, CD4^+^ CD103^−^ Trm represent the main pool and is located in the dermis (94, 99, 100). Little is known about the mechanisms controlling Trm residency and maintenance within tissues. It has been shown that both environmental signal and transcriptional regulators instruct Trm differentiation from circulating effector T cells or potentially via an unidentified pre-committed precursor recruited via the epithelium (94, 95). While CD69 and CD103 are required for Trm development and maintenance, respectively, CXCR3 is required for Trm precursors entry in skin epithelium in the context of HSV infection (94). The role of CXCR3 has been recently confirmed in non-infected animals, where CXCR3 expression on preimmune mature CD8^+^ T cell (mature T lymphocytes that have not yet engaged with cognate antigen) controls their recruitment to the skin driven by CXCL10 (57). CXCR6 and CCR10 expression by CD8^+^ Trm is also required for their optimal formation or maintenance in the skin (58). CCR8 has also been demonstrated to be a marker of skin human Trm (59).

In addition to Trm, recirculating memory T cells (Trcm) has been identified in the skin (101, 102). As opposed to Trm, Trcm do not permanently reside in the skin but can re-enter the circulation and lymph nodes. These cells express intermediate level of L-Selectin and CCR7 required for lymph node homing in addition to E-selectin ligands addressing them the skin.

Skin resident cells also comprises Treg cells, most of them expressing CD103 and CD69 which maintain tissue homeostasis (62). DETC seeds the epidermis during fetal development in a CCR10 dependent manner and are then maintained in a CCR4 dependent manner *in situ* and not replenished from circulating precursors (103, 104).

At steady state, murine ILC1-3 populations were initially described as tissue resident cells (105) however, although extremely rare, circulating ILC1-3 have also been observed (about 0.01–0.3% of circulating human lymphocytes), most of them being ILC2 (106, 107). Intravital imaging of murine skin has shown that 50–100 CD103^+^ ILC2 per mm^2^ continuously patrol skin dermis with an average low speed comparable to the one of migratory DC (65). ILC2 promote proinflammation, epithelial repair and wound healing (108). ILC2 expand under pathologic condition such as allergen challenge (64). Although skin ILC2 increase has largely been attributed to local proliferation, whether ILC2 (or their progenitor) are recruited from the blood circulation to the skin by a cascade of molecular interactions, need to be specifically investigated. Indeed, recent evidences showed that ILC2 develop in the bone marrow from common lymphoid progenitor cells and egress for the blood circulation under IL-33 stimuli or pathogenic conditions to repopulate classical ILC2 niches such as the lung, skin, lymph nodes (106, 107).

## Skin lymphocyte positioning and trafficking at steady state

Recent accumulating evidences suggest that hair follicles (extending through the entire epidermis and terminating in the dermis) provide a conduit system for immune cells, regulating their migration from the epidermis to the dermis and *vice-versa*, by secreting distinct chemokines along the hair follicles (109). Furthermore, hair follicles have been involved in CD8^+^ and CD4^+^ Trm maintenance through chemokine and cytokine production (110, 111). Intravital microscopy experiments revealed that on resolution of infection, memory CD8^+^ were confined to the epidermis with a predominant slow-moving behavior, adopting a dendrite morphology and did not recirculate (112). This slow motility involved Rho-associated coiled-coil kinases (ROCK) and pertussis toxin (PTX)-sensitive receptors, but do not required CXCR3, CCR8, CXCR6, CCR10, or CD103 (58). Therefore, it remains to be determined which PTX-sensitive receptor principally controls the patrolling migration of skin resident CD8^+^ T cells. Interestingly, the location and behavior of memory CD4^+^ in the skin differ strikingly from memory CD8^+^, suggesting complementary roles of these two populations in cutaneous surveillance. Indeed, memory CD4^+^ are highly motile cells, localized in the dermis and recirculate (112). Hair follicles also represent a particular cutaneous niche for Treg which form clusters around follicles (63). Intravital 2-photons imaging of Foxp3-GFP mouse showed that Tregs are dominantly non-motile at steady state and are rarely found in the hypodermis but enriched in the dermis with ~2,000 Treg/mm^2^. A small proportion of Treg (<7%) have migratory behavior while 90% of effector CD4^+^ T cells were migratory at steady state (63). By contrast, B-cells, NK, and γδT cells where rarely found proximal to hair follicles (113).

## Lymphocytes in inflammatory and malignant skin

The crucial role of lymphocytes in cutaneous surveillance is highlighted by the increased frequency and severity of skin malignancies and infections in patients with immunodeficiencies and by the pathogenesis of various acute and chronic inflammatory skin disorders such as psoriasis or atopic dermatitis. Because lymphocytes exploit, at least a partially similar cascade of molecular interactions to home into the skin in various cutaneous pathologies and under homeostasis; our knowledge on cutaneous lymphocytes has potential to inform and benefit therapeutic intervention of these pathologies (Figure 2).

### Inflammatory conditions

Inflammation-driven lymphocyte recruitment into the skin use the same adhesion cascade described above which is boosted by the upregulation of adhesion/chemokine family protein expression both on lymphocytes and tissue side (Figure 2). Lymphocyte recruitment occurs mostly in post-capillary venules, where the shear stress is highly increased in comparison to lymph node high endothelial venules (114). At high shear stress (>6 dyn/cm^2^), tethers and slings which were firstly discovered in neutrophils, are required to stabilize cell rolling (115). Tethers are formed from pre-existing cell microvilli. When tethers detach and swing around the rolling cell, they can become self-adhesive substrates named slings. While naïve CD4^+^ T cells cannot form both tethers and slings at high shear stress, it has recently been shown that Th1, Th17, and Treg CD4^+^ T cell (but not Th2) form tethers and slings under inflammatory conditions (116). In response to pathogenic stimuli, cutaneous expression of homeostatic chemokines CCL27 (CTACK) and CCL17 (TARC) are upregulated and inflammatory chemokines are induced (36–38). Indeed, keratinocytes can produce chemokines and cytokines including CXCL9, CXCL10, CXCL11, CCL20, along with inflammatory cytokines tumor necrosis factor-α (TNF), IL-1α, and IL-1β, IL-6, IL-10, IL-18, and IL-33 (38). Furthermore, CCL27 is also induced down stream of pro-inflammatory cytokines such as TNF-α and IL-1 β (36). In a model of delayed type hypersensitivity, it has been shown that CLA and CCR4 expression are upregulated on lymph node T lymphocytes favoring their recruitment into the skin (48). In addition to CCR4, CCR10 has been shown to be upregulated at the surface of a small proportion of T lymphocytes in delayed type hypersensitivity and bacterial chancroid skin lesions but the latter did not contribute to skin recruitment in comparison to CCR4 which was more highly expressed (49). In contrast, CCR10 is highly expressed by psoriasis, atopic, or allergic dermatitis patient's infiltrating T lymphocytes inducing their recruitment to the skin and the chronic cutaneous inflammation (36). However, skin NK cells isolated from allergic contact dermatitis lesions do not express CCR10 or CCR4 but are instead guided by CXCR3, CCR5, and CCR6 (67). Similarly, dermal NK cells in acute psoriatic plaques express CXCR3, CCR5, CCR6 in addition to CCR8 and CXCR1, however they only express low levels of CCR4 and do not express CLA (68). Upon inflammatory conditions, the number of cutaneous Treg and the proportion of migratory Treg in the skin increase rapidly (63). This Treg recruitment and in tissue migration are P-selectin, E-selectin, and CCR4 dependent. Intravital microscopy experiments have also revealed that CD4^+^ T cell interstitial migration was αV dependent under diverse inflammatory conditions as opposed to steady state conditions where integrins are not involved (117). Further, pathogenic and inflammatory conditions modify matrix fiber composition and density in the skin, and may assist in immune cell entry (118). While these studies show heterogeneity in the mechanism of lymphocytes recruitment under inflammatory conditions, it is however interesting to note that the molecules involved are restricted to a short list. It is this same list of molecules that may therapeutically benefit recruitment of TILs into melanoma.

### Cutaneous melanoma

Melanoma is the deadliest and most common form of cutaneous cancer. It is also the more investigated cancer in terms of tumor/immune cell relationship due to its exceptional immunogenicity linked to a high mutational burden (119, 120). Studies investigating gene expression profiling of cellular composition of tumors firstly classified tumors in two main subsets: “inflamed” and “non-inflammed” (121). The “inflamed” subset is characterized by the expression of transcripts for chemokines, T- and innate immune cell-lineage specific markers and inhibitory molecules. The “non-inflammed” subset is characterized by transcripts for macrophages and angiogenesis specific markers. A recent study proposed a more complex classification describing 6 profiles based on immune cell composition across 30 type of cancers: “wound healing,” “IFNγ-dominant,” “inflammatory,” “lymphocyte depleted,” “immunologically quiet,” and “TGF-β dominant” (122). Most of cutaneous melanoma fall in the “wound healing,” “IFNγ-dominant,” “inflammatory,” and “lymphocyte depleted” categories. The “inflammatory” subset was associated to the best prognostic value. Thus, tumor-infiltrating lymphocytes (TIL) are generally associated with a favorable prognostic value across multiple cancers, including in primary melanoma (123–127). However, some studies reported no correlation (128–130). Historically TILs referred to lymphocytes who were directly at the contact with tumor cells and/or infiltrate tumor nest (131). In 1989, the following graded system has been adopted to classify TILs in primary melanoma as: “ABSENT” when no lymphocytes were present at all or not at the contact with tumor cells; “BRISK” when lymphocytes were present throughout the vertical growth phase or infiltrating the entire base of the vertical growth phase and “NON-BRISK” when lymphocytes were present in one or more foci of the vertical growth phase (124). In a larger (1241) cohort of primary melanoma patients, Weiss et al. show that only patients with brisk TILs correlates with improved overall survival and relapse free survival but not non-brisk and absent TILs (132). While TILs were first restricted to CD8^+^ cytotoxic T cells, nowadays, they englobe all immune cells observed in the tumor, comprising effector and regulatory T lymphocytes, B lymphocytes, and NK cells in primary melanoma (133). Furthermore, non-lymphocyte populations such as dendritic cells, myeloid-derived cells, macrophages, mastocytes also infiltrate tumors (134). Beyond their presence, TIL functional status highly impact the prognostic value and response to treatment, explaining the contradictory results mentioned ahead. It is now more evident that integration of measures of TIL infiltration, activation states, and phenotype (immune-suppressor, activator, exhausted, anergic) are required to predict treatment efficiency and disease progression. As examples, the presence of M1 macrophages, N1 neutrophils are associated with a favorable prognosis while the presence of M2 macrophages, N2 neutrophils, or Treg are pro-tumoral (135, 136). While TIL infiltration has a high impact on tumor progression, generally TILs poorly infiltrate most tumors due to strategies established by tumor cells to hijack immune cell afflux. Among them, tumor vessels can be irregularly distributed, disorganized, tortuous, dilated resulting in leaking vessels with high interstitial pressure due to the density of the surrounding tumor mass (137). This probably rends lymphocyte infiltration from blood circulation more difficult than in healthy tissue. Furthermore, the expression of P-Selectin and VCAM-1 is diminished on intratumoral vessels in comparison to non-tumoral adjacent vessels (39, 40). Angiogenic factors like VEGF, FGF, NO, or TGF-b induce ICAM-1, ICAM-2, VCAM-1, and CD34 downregulation on endothelial cells, rending them refractory to immune cell infiltration (41–43). Interestingly, another molecule involved in TILs infiltration is an alternative spliced variant isoform of CD44, CD44v10, expressed on TILs from melanoma patients inducing TILs migration in an selectin and integrin-independent manner (50). Finally, tumoral endothelial cells can express molecules which block T-cell infiltration like the endothelin B receptor and its ligands endothelin-1 (138). Chemokines involved in immune cell recruitment are also deregulated in melanoma. CXCR3 and CCR5 has been shown to be the major chemokine receptor involved in CD8^+^ T cell infiltration in melanoma (44, 45). CXCR3 along with CCR5 expression correlates with CD8^+^ T cell recruitment and favorable outcome in melanoma (51–54). Furthermore, it has been shown that a strong CXCL9 and CXCL10 expression within tumors correlate with high CD8^+^ T cell infiltration in patients with melanoma (46). It has recently been proposed that tumors can inhibit IFNγ-induced CXCL9 by secreting galectin-3, a lectin which block IFNγ diffusion by binding to the glycans of the extracellular matrix (139). The role of CXCR3/CXCL10 axis is not restricted to CD8^+^ T cell infiltration as it plays an additional role in NK cells, controlling both their infiltration inside tumors and their migration toward their targets (69, 70). While the adhesion molecules and chemokines that favor T cell infiltration in melanoma are known, the precise ones required for the infiltration of pro-tumoral immune cells such as M2 macrophages or Tregs need to be investigated with the intent to control the activation and effector functions of TILs within tumors. Toward this aim, it has been shown in a mouse model that M2 macrophages infiltration required CCR2 ligand expression in tumors (140). Chemokine receptors are also expressed by melanoma cells allowing them to disseminate elsewhere in the organism, preferentially in the draining lymph nodes, lung, liver, gut, and brain where they form secondary lesions (141, 142).

## Conclusion and future directions

Lymphocyte trafficking to and within the skin is a highly regulated process involving unique and complex combinations of adhesion molecules and chemokine receptors (Figure 2). These combinations are cell-specific thereby controlling the type and location of the recruited cells. Under inflammatory conditions, lymphocyte recruitment is boosted by the increased expression and/or induction of adhesion molecules and chemokines on both cells and tissue side (Table 1). Lymphocytes are effectively recruited in inflamed tumors, but they are inhibited by immunomodulatory mechanisms (Exhaustion, Treg infiltration). However, they poorly infiltrate non-inflamed tumors. CD8^+^ T cell infiltration in primary melanoma is of favorable prognosis which had lead researchers to mount strategies to boost immune cell trafficking into tumors (137). Mimicking strategies induced by an inflammatory context has shown some successes in cancer. Indeed, the upregulation of adhesion molecules and chemokines on tumor vessels induced by the selective delivery of TNF (NGR-TNF) to these vessels has shown to increase the recruitment of endogenous or adoptively transferred T cells (143). A second illustration of this success is the inhibition of VEGF/VEGFR by the use of inhibitory molecules or antibodies which has also shown to promote lymphocytes infiltration in the tumors (144, 145). As a last example of such mimicking strategy, the induction of inflammatory chemokines through the release of IFN-γ has shown to induced T-cell infiltration in tumors (146, 147). Despite these encouraging successes, future therapeutic strategies may selectively open tumor mass gates to CD8^+^ T cells while being refractory to Treg or suppressive myeloid cells entry. Uncovering the specific adhesion molecule/chemokine combination for each type of lymphocytes and manipulate them to select lymphocyte types allowed to reach the tumor site may help to improve immunotherapy in cancer. Indeed, similar to that recently performed in breast cancer, single cell RNAseq analysis of melanoma TILs could yield valuable insights into the precise guidance cues required for cutaneous tumor entry (92). Further, there are still gaps in knowledge of the prognostic value in identifying distinct populations within melanoma or how individual lymphocyte populations are altered by interventions such as checkpoint inhibition. In addition, we still fail to fully understand how innate and adaptive lymphocytes work together to influence cancer growth. Recently, NK cells were highlighted as major orchestrators of cDC1 recruitment into melanomas; an axis that provides promising potential (148). However, the role that innate lymphocytes play in the co-ordination of TIL recruitment and function is under studied. We believe that a better understanding of lymphocyte specific location and interacting partners would help future successes in melanoma treatment.

## Author contributions

FL and JG contributed to content conception, wrote, and revised the manuscript.

### Conflict of interest statement

The authors declare that the research was conducted in the absence of any commercial or financial relationships that could be construed as a potential conflict of interest.
